# Parallel multiplicity and error discovery rate (EDR) in microarray experiments

**DOI:** 10.1186/1471-2105-11-465

**Published:** 2010-09-16

**Authors:** Wayne Wenzhong Xu, Clay J Carter

**Affiliations:** 1 Minnesota Supercomputing Institute for Advanced Computational Research, University of Minnesota, Minneapolis, MN 55455, USA; 2 Department of Biology, University of Minnesota Duluth, Duluth, MN 55812, USA

## Abstract

**Background:**

In microarray gene expression profiling experiments, differentially expressed genes (DEGs) are detected from among tens of thousands of genes on an array using statistical tests. It is important to control the number of false positives or errors that are present in the resultant DEG list. To date, more than 20 different multiple test methods have been reported that compute overall Type I error rates in microarray experiments. However, these methods share the following dilemma: they have low power in cases where only a small number of DEGs exist among a large number of total genes on the array.

**Results:**

This study contrasts parallel multiplicity of objectively related tests against the traditional simultaneousness of subjectively related tests and proposes a new assessment called the Error Discovery Rate (EDR) for evaluating multiple test comparisons in microarray experiments. Parallel multiple tests use only the negative genes that parallel the positive genes to control the error rate; while simultaneous multiple tests use the total unchanged gene number for error estimates. Here, we demonstrate that the EDR method exhibits improved performance over other methods in specificity and sensitivity in testing expression data sets with sequence digital expression confirmation, in examining simulation data, as well as for three experimental data sets that vary in the proportion of DEGs. The EDR method overcomes a common problem of previous multiple test procedures, namely that the Type I error rate detection power is low when the total gene number used is large but the DEG number is small.

**Conclusions:**

Microarrays are extensively used to address many research questions. However, there is potential to improve the sensitivity and specificity of microarray data analysis by developing improved multiple test comparisons. This study proposes a new view of multiplicity in microarray experiments and the EDR provides an alternative multiple test method for Type I error control in microarray experiments.

## Background

The microarray has become an important platform for a variety of bioscience and medical research areas. It allows researchers to detect the expression of thousands of genes simultaneously and to identify the differentially expressed genes (DEGs) based on statistical analysis of sample comparisons. However, due to the large number of tests that are performed, there are anticipated errors in identification of DEGs, and it is important to compute the error rate. This information aids in the initial evaluation of the discovery and also reduces the cost of validation experiments.

To date, many different multiple test methods that detect overall Type I error rates have been used to interpret microarray experiments [1-3]. The original multiple test methods were created for comparisons that were carried out many times simultaneously. For example, to test a drug's effects on several groups of subjects [4], one wants to report the probability of at least one null hypothesis (the family-wise error rate, FWER) in the side effects of this drug. FWER (α*) is derived from the total number (*m*) of *p-*values at the significance level (*α*), e.g. α* = 1 - (1 - *α*)^*m *^[5]. All tests are subjectively dependent, *i.e. *all *p-*values are related as a whole or as a single subject, for example the side effects. All test *p-*values must be used in this multiple test method. In microarray experiments, *p-*values are generated for tens of thousands of genes. Each *p-*value has no meaning with regard to other *p-*values, and all *p-*values are not related as a whole or to a single subject even though subgroups of genes exhibit biological dependency to some extent. Therefore the rationale of an FWER derived from the total gene number would need further evaluation in microarray experiments. For example, it may not matter if one is certain (probability of 1.0) that a gene list contains one or two false positives, *i.e*. FWER is 1.0. The common criticism of FWER is that it is too stringent and that it lacks power because FWER increases exponentially with the number of tested hypotheses [1].

A commonly used multiple test alternative to FWER, False Discovery Rate (FDR) is defined [6] as the expectation (*E*) of false positive genes (*V*) among selected genes (*R*), *i.e*. FDR = *E*(*V*/*R*). While FDR is conceptually appealing, there is no way to identify the number of actual false positives *V*. Multiple test procedures thus estimate the proportion (*p*_0_) of null hypothesis genes and multiply it by the total gene number and a false cutoff (*α*) [7]: *V *= *p*_*0*_.*m*. *α*. There are several variations of FDR methods [8], but the main difference is in the method of estimating this *p*_0_. Thus, all of these FDR methods are still constrained by the total gene number on the array, similar to the use of an FWER. The permutation approach for FDR counts the times that occur at a lower *p-*value than the actual *p-*value [9]. An ideal FDR method should present the false discovery rate within a given gene list. It is apparent, however, that permutation deduction does not directly reflect the false rate within the genes selected. Also, it possesses low power for microarray experiments that have a small number of sample replicates. The Bayesian model-based algorithm for FDR relies on a *prior *probability calculation [10], however, in real cases, this *prior *determination is biased.

Previously reported multiple test methods are constrained by the total gene number used. Problems arise when an experiment uncovers only a few DEGs among a large number of total genes. These few but real positive genes result in a very high FDR and therefore could be eliminated from the resultant DEG list. Because of this problem, reduction of the total gene number by gene filtering was reported to increase the power of the multiple testing [11]. This approach is questionable because one could eventually reach a lower FDR with continued filtering. The total gene list should not be filtered except for reasons of poor quality or violation of the multiplicity concept.

Researchers are often confused by the availability of so many different multiple test procedures. Thus they typically try several procedures at the same time and only report the most satisfying one, or they try to filter genes until they reach a satisfying result. It is apparent that with these approaches, the results from different research reports may not be comparable. Here we contrast parallel multiplicity to the traditional simultaneousness concept, and propose a new multiple test called the Error Discovery Rate (EDR) for microarray experiments. This method overcomes the common problem of previous multiple test procedures where the Type I error rate detection has low power when the total gene number used is large and provides an alternative or standard Type I error rate method.

## Results

### Null hypothesis distribution and false discovery rate (FDR)

**Figure 1:**
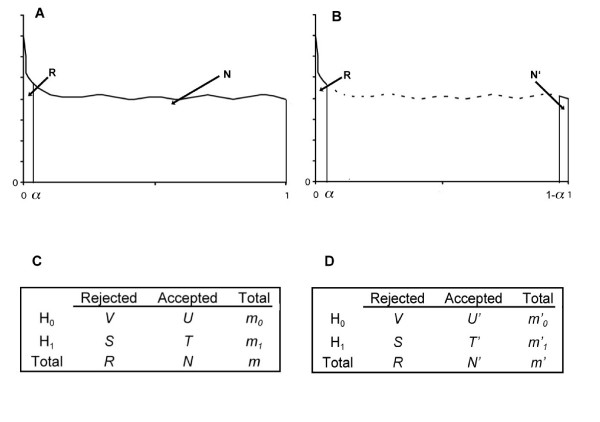
**Parallel multiplicity model versus simultaneous multiplicity model**. A and B indicate the *p-*value distribution with x-axis of *p-*values and y-axis of counts of test statistics. C and D are the simultaneous multiplicity tests and parallel multiplicity tests, respectively. H_0 _and H_1 _represent the null hypotheses and alternative hypotheses, respectively.

In a microarray experiment, one simultaneously conducts tens of thousands of individual gene hypothesis tests (*H*_*i*_),

$$H_{i}:\mu_{i}=0\;(i=1,...,m)$$

where *m *is the total number of genes targeted on an array and *μ*_*i *_is the mean log ratio of expression levels for the *i*th gene. These tests produce *p*-values (*p*_1_,...,*p*_*m*_) for all genes *m*. At a certain significance level *α*, one can define all genes into two groups: one is called "rejected null hypotheses" (*R*, Figure 1) with *p*-values equal to or less than *α *and containing the significantly changed genes or positive genes we actually observed; and the other group is called "accepted hypotheses" (*N*, Figure 1) with *p-*values higher than *α *and encompasses the non-significant genes or negative genes we actually observed.

If the *p*-value densities are plotted (Figure 1A), typically the negative gene *p-*values (*N*) are under a flat curve indicating that the null tests follow a uniform distribution. The positive gene *p-*values (*R*) close to a *p-*value of zero is higher than the flat region of negative gene *p-*values (*N*).

In these hypothesis tests, we are interested in the Type I error rate in the resultant positive genes *R*. However, only *m*, *R*, and *N *can be seen among all other parameters (Figure 1C) in these tests. The ideal way to find the errors (*V*) within *R *is by using experimental validation such as quantitative RT-PCR to determine the false positives. However, such an experimental approach is impractical for hundreds or thousands of *R *genes. Indeed, some studies randomly choose a few DEGs for qRT-PCR and report the ratio of number of confirmed DEGs to number of examined DEGs as FDR. Statistically, it can be assumed that the null distribution provides the desired control under the true or alternative distribution [1]. Therefore, the known parameter *N *or *m *can be statistically used for *V *approximation.

In the simultaneous multiplicity model, the proportion of unchanged genes (*p*_0_) among all genes (*m*) is estimated in order to deduce the total null genes (*m*_0_, Figure 1C). Thus, a natural estimate of FDR (*t*) when all null hypotheses are rejected with *p*_*i *_≤ *t *is

(1) $$FDR(t)=\frac{m_{0}t}{R(t)}$$

The key is that simultaneous multiple tests use the total unchanged genes (*m*_0_) for the error estimate. To date, much effort has been undertaken for the *m*_0 _determination. Storey 2004 [12] applied a tuning parameter, *λ *∈ [0,1), for the *m*_0 _estimate (*m*_0 _(*λ*)). Other methods, including nonparametric and parametric methods, have also been proposed to estimate *m*_0_, including the beta-uniform model [13], the spacing LOESS histogram [14], the Lowest Slope estimator [15], the smoother [16], the bootstrap least squares estimate [7], the successive elimination procedure [17], the moment generating function [8], and the Poisson regression approach [18], among others. All of these FDR methods estimate null hypotheses (*m*_*0*_) by applying various parameters on *p*-values of the total number of genes *m*.

### Parallel multiplicity and error V estimate

A new idea for multiplicity is proposed here that tens of thousands of genes can be tested in parallel in the microarray experiment. This "parallel" approach is different from the many "simultaneous" approaches that occur in the literature [1-3] and that introduce multiple tests into microarray experiments. Parallel multiplicity is composed of two complicities of the microarray: the normalization and the controls. Data normalization places all genes to be objectively or observationally related. The unchanged genes as negative controls influence the reliability of error detection. For example, assuming that the *p-*value reflects the error probability of a gene that is claimed as a positive gene, if a gene has 0.01 of error (a *p-*value of 0.01), the context of this error would be different (adjusting *p-*value or error discovery rate) when there are many negatives as controls in parallel versus when there are no controls in parallel. We prove how these negatives that parallel the positives can be used in error estimation.

#### LEMMA 1

*The error estimation (N'. t*) *by using the number of null tests (N') that have p-value 1-t and above is close to the real error (V) at the same significance level of t*.

##### Proof

Let *φ *be the error rate in a selected gene list *R*, and the real error *V *= *R. φ*. Let *V*_(__*m*__0) _be the error estimated at *p*_*i *_≤ *t *using *m*_0_, then *V*_(__*m*__0) _= *m*_0_.*t*. Let *V*_(__*N*__') _be the error estimated at *p*_*i *_≤ *t *using parallel negatives that have *p-*value 1- *t *and above, then *V*_(__*N*__') _= *N'*.*t*.

Assume that *m*_0 _>>*R *(this is also the assumption for microarray data normalization). Under this normalization assumption, we assume that *N*' ~ *R *and *φ *~ *t*. Then

$$V_{\left(m0\right)}-V=m_{0}.t-R.\varphi >>0$$

$$V_{\left(N^{'}\right)}-V=N^{'}.t-R.\varphi \approx 0$$

For those null tests (*x*) that have any *p*-value interval bins less than 1-*t*, the number of *x *may be the same as *N' *since the null *p-*values are of uniform distribution [19]. For example, the number of *p-*values between 1 and 1-*t *is the same as the number of *p-*values between 1-*t *and 1-2*t*. However, the context of these null tests is different. The null tests having higher *p-*values have higher probability of being true nulls, and the largest *p-*values are most likely to come from the true null, uniformly distributed *p-*values [7]. For example, a gene with *p-*value of 0.9 will have a 90% chance to be a true null test. The parallel idea uses the strongest contrastingly related (positives versus negatives) gene sets to control the Type I errors even though other portion of null tests might also be used.

### Error discovery rate (EDR)

In this parallel multiplicity idea, the parallel negatives (*N'*) at a certain significance level (*t*) are used to determine the error *V*. Therefore the error discovery rate (EDR) in a selected gene list (*R*) is defined as

(2) $$EDR(t)=\frac{N^{'}.t}{R(t)}$$

It can be seen that the contrast of EDR to FDR is that FDR uses total unchanged gene *m*_0 _but EDR uses the parallel negative genes *N' *to approximate error rate. Since the expression of most genes in microarray experiments is unchanged (the data normalization assumption), the *m*_*0 *_is close to *m*. In cases where the *m *is large and *R *is small, the FDR in equation (1) would pose a problem, *i.e. *the small number of real DEGs could be eliminated because it results in a high FDR. That is the reason for the use of the gene filtering approach to decrease the total gene number *m *in order to increase the detection power. However, in the EDR method, the positives (*R*) and negatives (*N'*) are sampled at each significance level (*t*) (Figure 1B and 1D) from all genes (*m*) and the distribution of the *p*-values. It can be seen in equation (2) that EDR would not pose a problem even when there is a large *m *and a small *R*, hence no gene filtering is needed for EDR.

The EDR in equation (2) is not an estimate of the probability of a DEG to be discovered in error. It is generally lower than the latter because it is computed using all the genes that have lower *p-*values than gene *i *(*p*_*i*_). It is apparent that a gene whose *p-*value is near the threshold *t *does not have the same probability to be differentially expressed as a gene whose *p-*value is close to zero. The EDR gives too optimistic a view of the probability for the gene to be an error. Thus, an EDR attached to each gene *i *that has *p*-value (*p*_*i*_) was defined by removing the denominator *R*_(__*t*__)_

(3) $$ED{R^{'}}_{i}=N^{'}.p_{i}$$

This is similar to the local FDR proposed by Aubert *et al*. [20] that is derived from the q-value estimation

(4) $$\bar{FDR}(i,\lambda )=m_{0}(\lambda )(p_{i}-p_{i-1})$$

The difference between equation (3) and (4) is that local EDR replaces *m*_0_(*λ*) and (*p*_*i *_- *p*_*i-*__1_) of local FDR by using *N*' and *p*_*i*_, respectively. As noted above, *N' *is different from the *m*_0 _(*λ*). Compared to (*p*_*i *_- *p*_*i*__-1_), *p*_*i *_retains more of the original test statistics of gene *i*.

Further, a reality error parameter *σ *is applied to the EDR model. Let $\sigma_{i}=\frac{1}{x_{i}(f_{i}-1),}$ where *x*_*i *_is the ratio of the maximum group mean of gene *i *with the median value of genes of all groups. *f*_*i *_is the fold change between groups of gene expression *i*. For example, each group of genes gives an expression mean value, and a maximum mean value is given from these group means. *x*_*i *_produces a reliability factor of the gene expression values. The fold change is log transformed for both up-regulated and down-regulated fold changes. When *f*_*i *_is equal to 1.0, there is no change in this gene expression between groups. Clearly, a small *x*_*i *_with small *f*_*i *_would lift the error. Applying these reliability factors to adjust the error rate is especially important in cases where only a few sample replicates are used because of the unstable variance that arises when sample size is small.

The final error discovery rate of gene *i, i *= (1,..., *m*), is derived from the number of negative genes (*N*') that parallel to gene *i *at a *p-*value of *p*_*i*_:

(5) $$EDR_{i}=N^{'}p_{i}\sigma_{i}$$

The EDR method can be implemented for two-group statistic tests (Additional File 1) or multiple-group ANOVA tests with gene expression matrix file, in which the *f*_*i *_is the fold change between the group with highest expression and the group with lowest expression of gene expression *i*. The EDR method can also be implemented with only *p*-values available as in equation (3).

### Case studies

In order to explore the problems or behaviors of multiple testing methods in real experiments, we chose three real case data sets that typically represent three situations: one contains a small number of DEGs, one has a moderate number of DEGs, and one presents a large number of DEGs.

#### Hyperinsulinemic data

This data set was used to compare the method powers in the real case that the proportion (*S*_0_) of differentially expressed genes is extremely low. This hyperinsulinemic data set was reported in a study to examine the effects of insulin on gene expression in healthy humans [21]. The original studies only reported three DEGs (GOS2, TXNIP, and BCL6). The EDR method found five DEGs among the 46 genes that passed the raw *p-*value cutoff at the significance level of *α = *0.05; while other FWER, FDR, SAM, and Bayes methods could not find any positive genes at the same significance level (Table 1) including the previously reported GOS2 gene that has a very low raw *p*-value (0.00018) and a high fold change (5.3). The five DEGs detected by EDR (Table 2) include GOS2, but not TXNIP and BCL6 that were in the original report [21]. This may result from a different data preprocess as these two genes, TXNIP and BCL6, have raw *p-*values of 0.26 and 0.10 from the two-tailed t-test, respectively. The evidence that the DDX5 gene is differentially expressed in diabetic mice [22] suggests that DDX5 detected by EDR in this study may be a true DEG.

**Table 1:** Differentially expressed gene numbers reported by different multiple tests.

	Data sets		
	
Test	Hyperinsulinemic	miRNA knockout	Colorectal cancers
raw p	46	2350	10973
PCER	104	4351	18425
PFER	9	442	2025
Bonferroni	0	144	605
Holm	0	144	609
Hochberg	0	144	609
SidakSD	0	144	614
BH	0	407	5552
BY	0	227	2221
qvalue	0	407	6108
SAM	0	0	5330
Bayes	0	0	5705
EDR	5	593	4810

The raw cel files of these three data sets [21,23,25] were downloaded from the NCBI GEO database (GSE7146, GSE7333, GSE4107) and were preprocessed by the GC-RMA method. Two groups in each data set were tested by two-tailed t test assuming equal variance. All multiple tests and raw *p-*values were applied at the same significance level of *α *= 0.05.

**Table 2:** EDR calculation

id	Gene	raw-*p*	***x***_***i***_	***f***_***i***_	*N*'	EDR
M72885_rna1_s_at	GOS2	0.00018432	309.453094	5.318301	2	0.00000028
X15729_s_at	DDX5	0.00044674	2.773653	1.332583	4	0.00193717
M34516_r_at	IGL@	0.00321646	10.316779	1.807361	27	0.01042631
D11428_at	PMP22	0.00342428	20.312435	1.910159	30	0.00555663
HG3514-HT3708_at	Tropomyosin	0.00437053	41.545886	1.188196	33	0.01844629
L20971_at	PDE4B	0.00494670	3.348770	1.998387	42	0.06214142
U33448_s_at	LTB4R	0.00625588	0.582200	1.066264	53	1

EDR detection of the Hyperinsulinemic data set. The EDR of gene *i *is the expectation of raw-*p *of this gene (*p*_*i*_) multiplied by the number of negative gene controls (*N*') at *p-*value equal to or greater than 1- *p*_*i*_, divided by the ratio of the maximum group mean of this gene with the median value of all genes (*x*_*i*_) and by the fold changes (*f*_*i*_) minus 1.

#### miRNA knockout data

**Figure 2:**
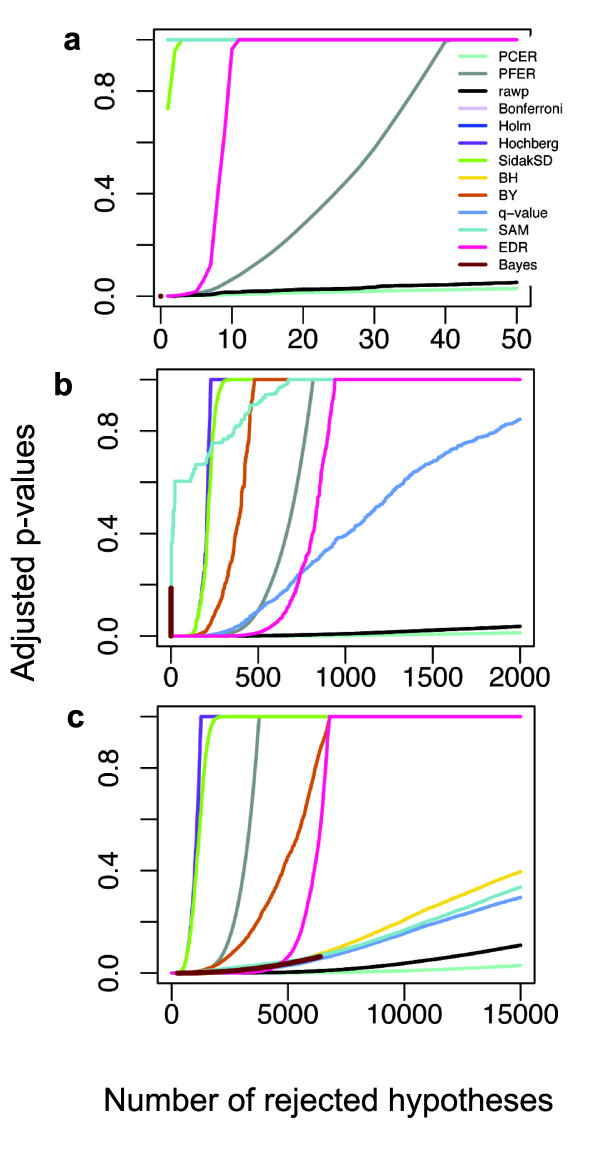
**Performances of all multiple tests on three different real data sets**. (a) low-*S*_0 _(proportion of changed genes) hyperinsulinemic data. (b) moderate-*S*_0 _miRNA knockout data. (c) high-*S*_0 _colorectal cancer data.

This data set [23] was used to compare the methods in the real case that *S*_0 _is in the moderate range (several hundred DEGs). Specifically, this miRNA knockout data set was reported from an examination of the effects of miRNA on gene expression in mouse heart tissue using the miRNA knockout model [23]. This miRNA deficiency would induce quite a number of gene expression differences [24] even though this study only focused on 70 protein-coding genes differentially expressed in miR-1-2-null hearts. In this data set, the EDR method caught more positive genes than any other multiple tests except PCER. The PCER method identified more genes, but its gene number is even higher than the number found by the raw *p-*value cutoff (Figure 2B, Table 1).

#### Colorectal cancer data

The data set examined [25] was used to compare the methods in a real case with a large *S*_*0 *_(several thousand DEGs). This data set of colorectal cancers was reported from a systematic search for genes differentially expressed in early-onset colorectal cancers using the GeneChip U133-Plus 2.0 that contains 54675 probe sets on Array [25]. In the original study, 9628 probe sets were found to be differentially expressed between patients and healthy controls according to two-tailed t-test analyses with *p *< 0.05. When the data set contains a large proportion of DEGs, EDR still detected more DEGs than FWER and BY but becomes slightly more stringent than other FDR methods such as SAM and Bayes (Figure 2C, Table 1). This appears sensible in real microarray experiments where, when there are so many positives, one strives for more stringent selection.

In these three real case data tests, it was found that the FWER and FDR methods were unable to detect DEGs at a significance level of 0.05 when only a small number of DEGs exist because of the high error rates that resulted. At the same significance level, the EDR method not only detected DEGs when only a few DEGs existed, but also caught more DEGs than FDR and FWER methods when there existed a moderate number of DEGs. Interestingly, EDR becomes more stringent when there are a large number of DEGs. However, since the DEGs were not validated in these experiments, we cannot determine whether the increase in the number of DEGs is due to an increase in power or an increase number of false DEGs. Hence, the specificity and sensitivity of these methods were further evaluated.

### Specificity and sensitivity

In order to evaluate specificity and sensitivity, one needs to know the "true" DEGs and "false" DEGs. As such, a published microarray expression data set [26] complemented with cDNA sequence digital expression confirmation was used to test the specificity and sensitivity of EDR and other multiple test procedures. The same RNA samples from Mexican axolotl animals were examined by *Ambystoma *GeneChip and 454 cDNA sequencing. DEGs between 0 and 5 days post amputation of denervated (DL) forelimb tissues were detected by microarray analysis at different significance levels. The resultant DEGs were compared to the true DEGs (true positives, TP) that were found to be differentially expressed via cDNA sequence digital expression analysis, the false DEGs (false positives, FP) that were not found differentially expressed via direct cDNA sequencing, and the true negatives (TN) that were not differentially expressed in both platform detections.

**Figure 3:**
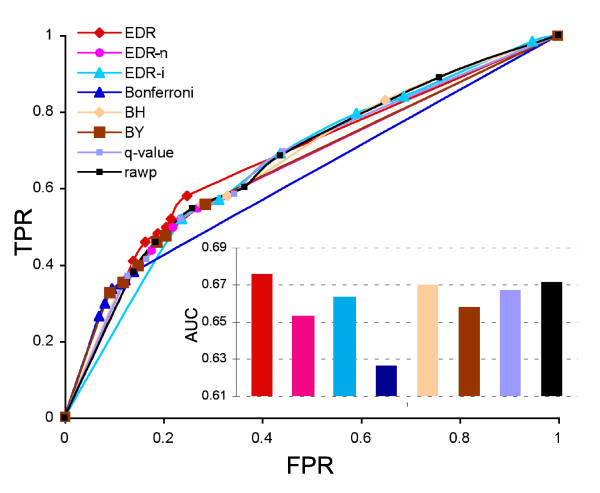
**Receiver operator characteristic (ROC) curve**. The true positive rate (TPR) and false positive rate (FPR) in differentially expressed genes (DEGs) detected by EDR [equation (5)], EDR-n [equation (3)], EDR-i [equation (2)], or other methods were plotted as ROC curves. The microarray data set [26] tested was confirmed by sequence digital expression.

The true positive rate (TPR) and false positive rate (FPR) of EDR and other methods at the significance of 0.05 are shown in Table 3. It can be seen that the TPR and the FPR levels are a trade-off. For example, EDR had better TPR (0.5) than Bonferroni (0.35) and BY (0.46), but higher FPR (0.2) than Bonferroni (0.11) and BY (0.19). However, when TPR and FPR were plotted on the Receiver Operator Characteristic (ROC) curve, the EDR curve was plotted above all other methods, and approached the left-top corner where the highest TPR and the lowest FPR exist (Figure 3). This indicates that EDR had an overall better accuracy in performance over other methods. The area under the curve (AUC) of EDR was the highest (0.676).

**Table 3:** TPR and FPR at significance of 0.05

			**Methods**			
	
	**EDR**	**Bonf**	**BH**	**BY**	**qvalue**	**rawp**
	
DEGs	1070	606	1420	974	1734	1833
TP	90	63	101	83	106	109
FP	980	543	1319	891	1628	1724
TN	3774	4238	3424	3870	3110	3011
FN	91	118	80	98	75	72
TPR	0.4972	0.3481	0.5580	0.4586	0.5856	0.6022
FPR	0.2061	0.1136	0.2781	0.1871	0.3436	0.3641

The expression data set was downloaded from http://www.ambystoma.org and was preprocessed by the RMA method [38]. Differentially expressed genes (DEGs) were detected at the significance level of 0.05 by the EDR method and the other methods from the multtest package [39]. The resultant DEGs were compared with the true DEGs (TP) measured by digital expression. False positives (FP) are those DEGs that are not found to be differential in digital expression analysis. True negatives (TN) are those genes that are not differential in both platforms.

It should be noted that in this microarray data set, the total number of gene probe sets on the array was small (4844), and it has a moderate number of DEGs (several hundred). This is an optimal case for simultaneous multiplicity model methods. Even in this case, EDR exhibited slightly improved performance over other methods. Therefore it may be speculated that the superior performance of EDR would likely be most evident in cases with large numbers of genes on the array and fewer DEGs.

### Simulation study

**Figure 4:**
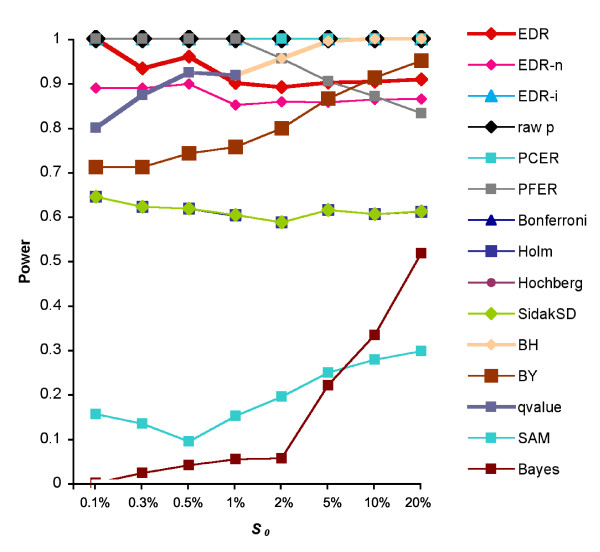
**Power comparison**. Power comparison of all multiple tests on simulation data sets with different proportions of differentially expressed genes. All FWER methods have the same powers on the same line.

To test the above speculation that EDR would have a better performance over other methods when a small number DEGs exist among a large number of genes on the array, existing Mouse GeneChip data [23] was simulated with a different proportion (*S*_0_) of changed genes (Additional File 2) and the powers of all multiple test methods were compared at different situations varying in DEG number. The power is defined as the expected proportion of true DEGs that are declared as DEGs [27]. The simulated DEGs were assumed as the true DEGs for power estimation [2,8,11,12,27-29]. As expected, only when a higher proportion of genes are differentially expressed (*S*_0 _is high) can all FDR tests increase power (Figure 4). All FWER methods have low powers and their powers do not increase as *S*_0 _increases. Even though the powers of SAM and Bayes increase when *S*_0 _increases, they still hold the lowest powers. However, at the same significance level of *α *= 0.05, EDR detected all simulated positive genes when *S*_0 _is 0.1%. When *S*_0 _is less than or equal to 0.5%, EDR had more power than the commonly used FDR methods BY and BH. When *S*_0 _became larger, EDR caught slightly fewer DEGs but still retained consistently high power. This is particularly useful when there are so many 'DEGs' that a more stringent selection is needed.

The power of EDR is derived from the error estimate from *N*'. As shown in Figure 4, without the reality factor, the EDR_*n *(*p*_*i*_.*N*') of equation (3) had more power than the commonly used method HY and BY when the DEG number is small. After applying this reality factor, the EDR (*p*_*i*_.*N*'.*σ*_*i*_) power of equation (5) was further improved.

It can also be seen that at the significance level of 0.05, the raw *p*-value, PCER, as well as the EDR-*i *of the equation (2) ($\frac{N^{'}t}{R(t)}$) detected all simulated DEGs. Their powers reached the maximum of 1.0 or more at different *S*_0 _points. Obviously, examining only power cannot determine the performance of these methods because these methods may have included many false DEGs as well.

Since these simulation data sets contain a large number of non-DEGs (true negatives, *TN*), the ROC curve would be skewed to the very low false positive rate ($FPR=\frac{FP}{FP+TN}$) side. We calculated the Precision-Recall (PR) curve instead. While the PR and the ROC are equivalent, the PR curve is more informative when dealing with highly skewed data sets [30]. When there is a small *R *(*S*_*0*_: 0.001) and big *m *(45101), the EDR family exhibited better performance than Bonferroni and BY, but had a similar AUC size to BH and rawp methods (Additional File 3A). Because the error rates of all multiple test methods are derived from raw *p*-value and the lower raw *p*-value is equivalent to an FDR or EDR, it is not surprising that the rawp has a large AUC among all three data sets (Additional File 3A, B, C). However, a closer examination of the rawp curve in Additional File 3A showed that several cutoffs of rawp give very low precision, indicating false DEGs were contained in the detected DEGs. The significance level of 0.05 is commonly chosen for statistics and for power evaluation [11,15,29,31-33]. At this significance level, EDR had a better precision (simulated true DEGs among all detected DEGs) than BH and other methods (Additional File 3A, lower panel). Even though the EDR-*i *and rawp caught more simulated true DEGs, *i.e. *a higher power as showed in Figure 4, their precisions were relatively low. When there were several hundred DEGs (Additional File 3B), EDR exhibited better performance than Bonferroni and BY, but lower than BH (Additional File 3B, upper and middle panels). When the simulated true DEGs go up further (Additional File 3C), EDR became more stringent than other methods except Bonferroni.

## Discussion

There are many research papers and reviews on multiple testing for microarray experiments [1-3]. Most of the literature discusses how to apply various multiple tests in microarray experiments, but the theory of multiplicity for the microarray has not been depicted. General statements are found where "as a typical microarray experiment measures expression levels for thousands of genes simultaneously, large multiplicity problems are generated" [1]. The parallel multiplicity concept proposed here stands in contrast to the traditional "simultaneous" multiplicity. Parallel multiple tests use only the negative genes that parallel the positive genes to control the error rate while simultaneous multiple tests use the total unchanged gene number for error estimation even though both the negative gene number and the total unchanged gene number depend on the total gene number and the distribution of the *p*-values. The EDR method based on parallel multiple tests exhibits improved performance over simultaneous multiple test methods in specificity and sensitivity. Since parallel multiple tests use only the negative genes that parallel the positive genes, not the total unchanged genes, the EDR method overcomes the common problem found in previous simultaneous multiple test procedures where the Type I error rate detection power is low when the total gene number used is large and the DEG number is small. It is a robust method for a variety of statistical *p-*value distributions [34]. EDR retains the power for various data sets without requiring gene filtering as well.

Specificity and sensitivity are crucial criteria for method comparison, and the true and false positives need to be known for these tests. However, there is no gold standard to determine true or false positives. There are three levels for the definition of "true": true hybridization signal changes, true mRNA transcript changes, and true functional changes. True hybridization signals can infer mRNA transcript levels (gene expression levels), but may not necessarily represent the true mRNA transcripts, and the true mRNA transcript changes may not cause the gene functional changes within an organism. Here we focus only on the expression level, *i.e. *the mRNA transcript level. To date, many microarray multiple test studies use simulation data or case data to evaluate the power (sensitivity) and specificity [2,11,35]. Those microarray data sets with very few qRT-PCR validations are not useful for evaluating the specificities of multiple tests. In this study, a Mexican axolotl animal microarray data set with cDNA sequence digital expression validation [26] was found to be useful. This data set possesses a relatively large number of "true" and "false" positives determined at mRNA transcript levels. Even though 454 sequencing also has a certain level of false positives, the comparison using this same data set for EDR and comparison of other methods is compatible. In testing this data set, the EDR method exhibited slightly improved performance over other methods in specificity and sensitivity. This improved performance is not as remarkable as in other cases, because this data set, with a low number of genes on array and a moderate number of DEGs, is an optimal case for simultaneous multiple test methods. The consistent superior performance of the use of EDR was further supported by testing the simulation data and three real case data sets that vary in the proportion of unchanged genes.

Many reports use real microarray case data sets for multiple tests evaluations. For example, the acute lymphoblastic leukemia (ALL) data set was intensively used for such a purpose [2,8,20,35] even though there was no validation for the real DEGs in this data set. Other real case microarray data sets were also used for multiple test performance evaluation, including a breast cancer data set [16,20], a colon cancer expression data set [35], a wheat data set [11], a diabetes data set [11], and a smoking data set [11]. Not all of these data sets were validated for real DEGs. Three real case data sets were chosen for this study because they represent three typical cases: low, moderate, and high levels of DEGs. It is difficult to evaluate the individual method by unvalidated data, but these data sets are still helpful in comparing the behaviors or performance of different methods under the same circumstance.

The EDR model is different from the per-family error rate (PFER) and the per-comparison error rate (PCER) that simply adds individual *p-*values together or averages *p-*values [2]. It is distinct from FWER and FDR in that EDR utilizes cross-gene information in the parallel concept but is not constrained by the total gene number. It varies from permutation-based FDR in that EDR directly reflects the errors of the given genes and EDR operates for small sample sizes. Also, EDR is appealing because it attaches the error to each gene, and the genes do not need to be ranked by raw *p-*values as is the case for all other multiple tests. Other multiple test comparisons sequentially compute the error rate based on the ranked raw *p-*values; thus the error rates are the same as the lower *p-*values. This is not true in microarray multiplicity because at times lower *p-*values possess higher error potential than some higher *p-*values. EDR recognizes this reality (Table 2). There are indeed reports for how to rank and select genes [33], but they do not address the error rate issue.

A false positive or the rejection of a true null hypothesis is a gene that is called as differentially expressed when it is not. The falsehood or error can be a systematic error or random error. The systematic error can be corrected by background subtraction or dye normalization, while the random error cannot be captured but can be modeled by statistical analysis. The *p-*value of each gene is a measure of how much evidence we have against the null hypothesis. It is rational that all multiple tests use this raw *p-*value or marginal *p-*value as a base error rate of that gene [1-3]. Even though the *p-*value has inherently accounted for the fold change, an extremely small standard deviation (sd) could drive a small *p-*value with a small fold change. Almost all microarray studies apply a fold change cutoff for the final gene list to be reported. The reason that we only keep the genes whose fold changes are higher than a defined value, for example, a 2-fold change, is that it is still not certain that those low fold-changes in hybridization signals are real positives at the level of the mRNA transcript. After these uncertain genes are removed, the error rate in the final gene list would undoubtedly decrease. Also, some genes have very low intensity signals. For example, among the gene expression values ranging from 1.0 to 10,000, a 10-fold increase deduced from 10.0 divided by 1.0 is reasonable from a statistical point of view. However, biologists often remove this gene even though it has a very small *p-*value because this low expression level is close to background expression levels and therefore may be not reliable. Thus, an ideal multiple test procedure would incorporate into the raw *p-*values the unreliability factors that result from low intensity and low fold-change in the error calculation in order to reflect the error or false reality. In the EDR method, *x*_*i *_uses the maximum group mean and the median expression value to control the gene expression values, and uses *f*_*i *_to control the fold changes.

Different multiple test procedures were recommended for different experiments based on the sample size, the magnitude expectations, and the *p-*value distribution [34]. However, there may exist a large variation in judging these multiple parameters. Therefore the same type of experiments may produce different results depending on the method of preference among different researchers. There is not a comparable standard multiple test method that can be applied to a variety of data sets. It is interesting to note that the EDR method had a similar performance pattern in all three different case data sets (Figure 2). This pattern was not affected by the proportion (*S*_0_) of DEGs of the data. Also, this performance pattern presents a deep slope in all three data types. This deep slope forms a boundary useful for significance cutoff value selection. Other methods could not form this boundary in all data types. The performance patterns of other methods change vigorously depending on the *S*_0 _(Figure 2). For example, the BH performance curve did not show up in the low-*S*_0 _data set. It has lower power than PFCR in the median-*S*_0 _data set but was capable of much higher power than PFCR in the high-*S*_0_data set. This suggests that the EDR method exhibits consistent performance in various data sets. This consistency is further supported by the use of simulation data. The EDR method works for different data sets and would therefore provide a standard method for type I error rate calculations in microarray experiments. For example, we can say that with an EDR cutoff of 0.05 we found 100 differentially expressed genes between disease and normal samples. However, if we use other FDR methods, this may not be the case because some studies may find more genes using the same FDR cutoff in the same data set but by filtering genes or filtering in different ways, or they may use different FDR methods for data sets that vary in the proportion of unchanged genes.

It is difficult to test if the EDR method works for dependent tests in microarray experiments. The multiple-endpoint in clinical trials [36] is an example for FDR to control dependence in multiple tests. In this example, we know all tests are dependent. This knowledge is not deduced from *p-*values but instead by the experiment itself. In a microarray experiment, all *p-*values do not relate to one single subject, *i.e. *they are independent. Only the genes in subgroups may be dependent because they usually work in a collaborative fashion to fulfill certain cell functions or pathways. The FDR methods claim to be able to control the error rate of the dependent *p-*values, and simulated dependent data was used to test this claim [2,12,35]. Clumpy dependence was simulated in the sense that blocks of genes have dependent expression and therefore dependent *p*-values in *p-*value bins. However, this simulated dependence is quite different from the real microarray experiment. In a microarray experiment, the "correlated" *p-*values may not necessarily reveal true biological dependence, and true biological dependence may not exhibit dependence on *p-*value levels. For example, minor differential expression of some genes may play a remarkable regulatory function in a particular pathway, and the *p-*values of these genes may not be correlated to those of other genes in this same pathway. Further studies are needed to examine genes in the same pathways and then to adjust the error rate based on the same pathway groups.

## Conclusion

Microarrays are extensively used today to examine changes in gene expression. However, biologists have difficulty in both the understanding and use of multiple tests in microarray studies. A new system of parallel multiple tests is proposed here. Parallel multiple tests use the negative genes that parallel the positive genes to control the Type I error discovery rate (EDR) in the microarray experiment. The EDR method exhibits consistently improved specificity and sensitivity (power) over other methods in testing diverse data sets that vary in the number of null hypotheses. This method provides an alternative to standard Type I error rate methods. Parallel multiplicity is a new proposition and worth further enhancement in statistics and algorithm development.

## Materials and methods

### Hyperinsulinemic data and preprocessing

This data set was reported in a study to examine the effects of insulin on gene expression in human health [21]. Six nondiabetic volunteers were given a 3-h hyperinsulinemic (infusion rate 40 mU/m^2^/min) euglycemic clamp test. A variable infusion of glucose (180 g/l) was used to maintain euglycemia during insulin infusion. The gene expression profiles of skeletal muscle biopsies from these six subjects pre- versus post-clamp were compared using the Affymetrix GeneChip Hu6800 containing 7129 probe sets. Only three genes were reported to be regulated by insulin in human muscle cell using a Wilcoxon signed rank test after filtering removed 5952 probe sets.

The raw cel files were downloaded from the NCBI GEO database (GSE7146) containing data that are MIAME compliant as detailed on the MGED Society website http://www.mged.org/Workgroups/MIAME/miame.html The GC-RMA algorithm was used for probe level signal condensation, background subtraction, and normalization. The GC-RMA values were log-transformed for robust use in statistics tests since the log values are more compliant with the data normal distribution assumption than the raw data. But the fold and ratio calculations used the raw expression values. The raw expression values were truncated into 0.5% and 99.5% percentile ranges in order to avoid the extreme large and extreme small values in the fold and ratio calculations. With this data set, the EDR method was compared with 11 other multiple test procedures (Figure 2) at the same significance level of *α *= 0.05 (Table 1).

### miRNA knockout data and preprocessing

This data set was reported to examine the effects of miRNA on gene expression in mouse heart tissue [23]. Heart tissue samples from three wild type and three miR-1-2 knockout mice at postnatal days 10 were compared for gene expression levels using Affymetrix mouse genome 430 2.0 array that contains 45101 probe sets. The raw cel files were downloaded from the NCBI GEO database (GSE7333) and were preprocessed by GC-RMA algorithm. With this data set, the EDR method was compared with 11 other multiple test procedures (Figure 2) at the same significance level of *α *= 0.05 (Table 1).

### Colorectal cancers data and preprocessing

This data set was reported to systematically search for genes differentially expressed in early-onset colorectal cancers using the GeneChip U133-Plus 2.0 Array [25]. Twelve tumor specimens and ten adjacent grossly normal-appearing tissues from at least 8 cm away were collected for RNA extraction. The raw cel files were downloaded from the NCBI GEO database (GSE4107) and were preprocessed by GC-RMA algorithm. With this data set, the EDR method was compared with the other 11 multiple test procedures (Figure 2) at the same significance level of *α *= 0.05 (Table 1).

### Transcriptional validated data set

The data set used was reported in a study of transcription during nerve-dependent limb regeneration [26]. The same RNA samples were detected by *Ambystoma *GeneChip and 454 cDNA sequencing. There are total 4844 probe sets (TGs) on this GeneChip array.

The raw cel files were downloaded from the public Ambystoma Microarray Database [37]. Detailed information of these data files and the DEGs confirmation by 454 cDNA sequencing were described in the original study [26]. The RMA algorithm [38] was used for probe level signal condensation, background subtraction, and normalization. The RMA values were log transformed for robust use in statistics tests since the log values are more compliant with the data normal distribution assumption than the raw data. But the fold and ratio calculations used the raw expression values. Raw expression values were truncated into 0.5% and 99.5% percentile ranges in order to avoid the extreme large and extreme small values in the fold and ratio calculations. The two-tailed t test was used to calculate the raw *p*-values.

Of the DEGs detected among five group comparisons by microarray analysis, 271 DEGs were confirmed in mRNA levels by using 454 cDNA sequencing. In this evaluation, only two groups of raw data files, zero and five days post amputation of denervated (DL) forelimb tissues, were used. Among the total 271 true DEGs, 181 genes were true DEGs between DL0 and DL5 and possessed 1.5-fold or greater changes in normalized digital expression levels. At each significance level, the true positive DEGs (TP) were the portion of these 181 genes discovered by microarray analysis. The false positive DEGs (FP) were those total DEGs detected by microarray analysis (TDEGs) subtracted by TP. The false negative DEGs (FN) were calculated by subtracting TP from 181. The true negative DEGs (TN) were the TGs subtracted by TDEGs. Since 454 cDNA sequencing presumably covered all cDNAs or genes including all probe sets on the GeneChip, the portion of genes that were not detected by both platforms were TN.

### Simulation data

In order to calculate the statistical power of method, we simulated microarray data with different proportions (*S*_0_) of true positive gene numbers. The power was calculated as the proportion of true positives that were detected at the same significance level of *α *= 0.05.

The simulation parameters for gene group means (m_i_) and gene group variances (sd_i_) were replicated on the real miRNA knockout data set [23] and preprocessed by GC-RMA [38]. The simulated data matrix was created by 2 groups of 10 sample columns (n1 = 5, n2 = 5) and 45101 rows of genes (m = 45101) (Additional File 2).

Each gene expression value is generated within the normal distribution of mean m_i _and standard deviation sd_i_. m_i _is the gene group mean that is uniformly distributed within the minimum and maximum values of the whole miRNA knockout data set. sd_i _is the standard deviation of a gene group that is uniformly distributed within the minimum sd and maximum sd of all gene group sds of the whole real data set. The non-DEGs were guaranteed to have the normal distribution of the sd_i _and equal m_i_. also. In addition, the two group means are close to equal because if they fall into the normal distribution with an equal mean, they still may have large fold changes. This ensures they are non-DEGs in a statistical sense. The different *S*_0 _(0.001, 0.003, 0.005, 0.010, 0.020, 0.050, 0.100, 0.200) data sets were simulated by adding different proportions of DEGs. The normal distribution of gene expressions of DEGs in each group samples was simulated using mean and sds vectors. The mean vectors have folds uniformly distributed between 1.5- to 3-fold changes and the sds vector has uniformly distribution of one-fifth of the means (Additional File 2). For each simulation data set, two-group t-tests assuming equal variance were performed, and EDR and other multiple test methods were applied.

### Receiver operating characteristic (ROC) and precision-recall (PR) curves

For the transcriptional validated data set, the ROC curve of EDR method was compared with other multiple test procedures (Figure 3). The AUCs of ROC curves based on TPR and FPR were used to compare the overall performances of EDR and other methods. The curve approaching the left-top corner represents the highest TPR and lowest FPR.

For the false positive rate (FPR) skewed simulation data sets, PR curve was used instead for method performance comparison.

$$\text{TPR, Recall}=\frac{TP}{TP+FN}$$

$$\text{FPR}=\frac{FP}{FP+TN}$$

$$\text{Precision}=\frac{TP}{TP+FP}$$

### Multiple tests

The multiple tests for Type I error rates were categorized into several groups [2]: PCER, PFER, FWER, and FDR. The following tests are defined according to Figure 1D and used in this study.

#### *PCER *[2]

the per-comparison error rate is defined as the expected value of the number of Type I errors divided by the number of hypotheses, that is,

$$\text{PCER}=E(V)/m=\frac{a_{1}+...+a_{m}}{m}$$

#### *PFER *[2]

the per-family error rate is defined as the expected total number of Type I errors, that is, PFER = *E(V) *= *a*_1 _+ ... + *a*_*m*_

#### FWER

the family-wise error rate is defined as the probability of at least one Type I error (*a*_*i*_), that is, FWER = Pr(*V *≥ 1). The Bioconductor multtest package [39] was used for following PWER procedures: Bonferroni, Holm, Hochberg, and SidakSD.

Bonferroni [2], $p_{i}=\frac{\alpha_{i}}{m}$

Holm [40], *p*_*i *_= $p_{i}=\frac{\alpha_{i}}{m-i+1}$

Hochberg [41], *p*_*i *_= $p_{i}=\frac{\alpha_{i}}{m-i+1}$

SidakSD [42], $p_{i}=1-\sqrt[{m-i+1}]{1-\alpha }$

#### FDR

Bioconductor multtest, qvalue, and sam packages [39] were used for the following FDR detection (${\overset{ƒ}{p}}_{i}$): BH, BY, qvalue, SAM, and Empirical Bayes.

BH [6], ${\overset{ƒ}{p}}_{i}=p_{i}\frac{m}{i}$

BY [36], ${\overset{ƒ}{p}}_{i}=p_{i}\frac{m\sum 1/i}{i}$

qvalue [7], ${\overset{ƒ}{p}}_{i}=p_{i}\frac{m{\hat{\pi }}_{0}}{i}$, where ${\hat{\pi }}_{0}$ is the proportion estimation of unchanged genes.

SAM [9], ${\overset{ƒ}{p}}_{i}=\frac{\sum_{b}^{B}n_{p_{b}>p_{i}}}{B}$, where B is the number of permutations.

Empirical Bayes [10], $P(H_{0}/p\leq \alpha )=\frac{\alpha \pi_{0}}{\alpha \pi_{0}+(1-\beta )(1-\pi_{0})}$, where P is the FDR, *α *is the significance level, *π*_0 _is proportion of null hypothesis, and (1-*β*) is the test power.

## Authors' contributions

WX proposed the idea, performed the tests, and wrote the manuscript. CC contributed to the revision.

## Supplementary Material

Additional file 1 **R source code for EDR method**. The EDR method was implemented for two group comparison experiments either with *p*-values provided or without p-values. Click here for file

Additional file 2 **Simulation and parameters**. Existing Mouse GeneChip data [23] was simulated with a different proportion (*S*_0_) of differentially expressed genes. Click here for file

Additional file 3 **Precision-Recall (PR) curves**. Precision-Recall (PR) curves of multiple test methods on simulation data sets with different proportions of DEGs (A, *S*_*0*_: 0.001; B, *S*_*0*_: 0.02; C, *S*_*0*_: 0.2). Upper panel: PR curves; middle panel: the area under the curve (AUC); lower panel: simulated true DEGs (blue bars) contained within all detected DEGs of each method were at or below the significance level of 0.05. Click here for file

## References

[B1] Dudoit S van der Laan MJ Multiple Testing Procedures with Applications to Genomics Springer Series in Statistics 2008 Springer Science and Bussiness Media, LLC

[B2] Dudoit S Shaffer JP Boldrick JC Multiple hypothesis testing in microarray experiments Statistical Science 2003 18 71 103 10.1214/ss/1056397487

[B3] Farcomeni A A review of modern multiple hypothesis testing, with particular attention to the false discovery proportion Statistical Methods in Medical Research 2008 17 347 388 10.1177/0962280206079046 17698936

[B4] Gey GD Levy RH Fisher LD Pettet G Bruce RA Plasma concentration of procainamide and prevalence of exertional arrythmias Annals of Internal Medicine 1974 80 718 722 4832159 10.7326/0003-4819-80-6-718

[B5] van Belle G Fisher LD Heagerty PJ Lumley T Biostatistics A methodology for the health sciences 2004 2 John Wiley & Son, Inc

[B6] Benjamini Y Hochberg Y Controlling the false discovery rate: a practical and powerful approach to multiple testing J Royal Stat Soc Series B 1995 57 289 300

[B7] Storey JD A direct approach to false discovery rates J Royal Stat Soc Series B 2001 64 479 498 10.1111/1467-9868.00346

[B8] Broberg P A comparative review of estimates of the proportion of unchanged genes and the false discovery rate BMC Bioinformatics 2005 6 199 10.1186/1471-2105-6-199 16086831 PMC1199583

[B9] Tusher VG Tibshirani R Chu G Significance analysis of microarrays applied to the ionizing radiation response Proc Natl Acad Sci USA 2001 98 5116 5121 10.1073/pnas.091062498 11309499 PMC33173

[B10] Efron B Tibshirani R Storey JD Tusher VG Empirical Bayes analysis of a microarray experiment J American Stat Assoc 2001 96 1151 1160 10.1198/016214501753382129

[B11] Hackstadt AJ Hess AM Filtering for increasing power for microarray data analysis BMC Bioinformatics 2009 10 11 10.1186/1471-2105-10-11 19133141 PMC2661050

[B12] Storey JD Taylor JE Siegmund D Strong control, conservative point estimation, and simultaneous conservative consistency of false discovery rates: A unified approach J Royal Stat Soc Series B 2004 66 187 205 10.1111/j.1467-9868.2004.00439.x

[B13] Pounds S Morris SW Estimating the occurrence of false positives and false negatives in microarray studies by approximating and partitioning the empirical distribution of p values Bioinformatics 2003 19 1236 1242 10.1093/bioinformatics/btg148 12835267

[B14] Pounds S Cheng C Improving false discovery rate estimation Bioinformatics 2004 20 1737 1745 10.1093/bioinformatics/bth160 14988112

[B15] Hsueh H Chen JJ Kodell RL Comparison of methods for estimating the number of true null hypotheses in multiplicity testing Journal of Biopharmaceutical Statistics 2003 13 675 689 10.1081/BIP-120024202 14584715

[B16] Storey JD Tibshirani R Statistical significance for genome wide studies Proc Natl Acad Sci USA 2003 100 9440 9445 10.1073/pnas.1530509100 12883005 PMC170937

[B17] Scheid S Spang R A Stochastic downhill search algorithm for estimating the local false discovery rate IEEE Transactions on Computational Biology and Bioinformatics 2004 1 98 108 10.1109/TCBB.2004.24 17048385

[B18] Efron B Large-scale simultaneous hypothesis testing: the choice of a null hypothesis Journal of the American Statistical Association 2004 99 96 104 10.1198/016214504000000089

[B19] Fodor AA Tickle TL Richardson C Towards the uniform distribution of null P values on Affymetrix microarrays Genome Biol 2007 8 5 R69 10.1186/gb-2007-8-5-r69 17472745 PMC1929139

[B20] Aubert L Bar-Hen A Daudin JJ Robin S Determination of the differentially expressed genes in microarray experiments using local FDR BMC Bioinformatics 2004 5 125 10.1186/1471-2105-5-125 15350197 PMC520755

[B21] Parikh H Carlsson E Chutkow WA Johansson LE Storgaard H Poulsen P Saxena R Ladd C Schulze PC Mazzini MJ Jensen CB Krook A Björnholm M Tornqvist H Zierath JR Ridderstråle M Altshuler D Lee RT Vaag A Groop LC Mootha VK TXNIP regulates peripheral glucose metabolism in humans PLoS Medicine 2007 4 0869 0879 10.1371/journal.pmed.0040158 PMC1858708 17472435

[B22] Eckenrode SE Ruan Q Yang P Zheng W McIndoe RA She JX Gene Expression Profiles Define a Key Checkpoint for Type 1 Diabetes in NOD Mice Diabetes 2004 53 366 375 10.2337/diabetes.53.2.366 14747287

[B23] Zhao Y Ransom JF Li A Vedantham V von Drehle M Muth AN Tsuchihashi T McManus MT Schwartz RJ Srivastava D Dysregulation of cardiogenesis, cardiac conduction, and cell cycle in mice lacking miRNA-1-2 Cell 2007 129 303 317 10.1016/j.cell.2007.03.030 17397913

[B24] Krützfeldt J Rajewsky N Braich R Rajeev KG Tuschl T Manoharan M Stoffel M Silencing of microRNAs in vivo with 'antagomirs' Nature 2005 438 685 689 10.1038/nature04303 16258535

[B25] Hong Y Ho KS Eu KW Cheah PY A susceptibility gene set for early onset colorectal cancer that integrates diverse signaling pathways: implication for tumorigenesis Clin Cancer Res 2007 13 1107 1114 10.1158/1078-0432.CCR-06-1633 17317818

[B26] Monaghan JR Epp LG Putta S Page RB Walker JA Beachy CK Zhu W Pao GM Verma IM Hunter T Bryant SV Gardiner DM Harkins TT Voss SR Microarray and cDNA sequence analysis of transcription during nerve-dependent limb regeneration BMC Biology 2009 7 1 19 10.1186/1741-7007-7-1 19144100 PMC2630914

[B27] Lee MLT Whitmore GA Power and sample size for DNA microarray studies Statistics in Medicine 2002 21 3543 3570 10.1002/sim.1335 12436455

[B28] Wang SJ Chen JJ Sample size for identifying differentially expressed genes in microarray experiments Journal of Computational Biology 2004 11 714 726 10.1089/cmb.2004.11.714 15579240

[B29] Jung SH Bang H Young S Sample size calculation for multiple testing in microarray data analysis Biostatistics 2005 6 157 169 10.1093/biostatistics/kxh026 15618534

[B30] Xu WW Cho S Yang SS Bolon YT Bilgic H Jia H Xiong Y Muehlbauer GJ Single-feature polymorphism discovery by computing probe affinity shape powers BMC Genetics 2009 10 48 10.1186/1471-2156-10-48 19709416 PMC2746803

[B31] Jain N Thatte J Braciale T Ley K O'Connell M Lee JK Local-pooled-error test for identifying differentially expressed genes with a small number of replicated microarrays Bioinformatics 2003 19 1945 1951 10.1093/bioinformatics/btg264 14555628

[B32] Jiang H Doerge RW Estimating the Proportion of True Null Hypotheses for Multiple Comparisons Cancer Informatics 2008 6 25 32 19259400 PMC2623313

[B33] Yang YH Xiao Y Segal MR Identifying differentially expressed genes from microarray experiments via statistic synthesis Bioinformatics 2005 21 1084 1093 10.1093/bioinformatics/bti108 15513985

[B34] Pounds SB Estimation and control of multiple testing error rates for microarray studies Briefings In Bioinformatics 2006 7 25 36 10.1093/bib/bbk002 16761362

[B35] Wu B Guan Z Zhao H Parametric and nonparametric FDR estimation revisited Biometrics 2006 62 735 744 10.1111/j.1541-0420.2006.00531.x 16984315

[B36] Benjamini Y Yekutieli D The control of the false discovery rate in multiple testing under dependency Ann Statist 2001 29 1165 1188 10.1214/aos/1013699998

[B37] Ambystoma Microarray Database http://www.ambystoma.org

[B38] Wu Z Irizarry RA Gentleman R Martinez-murillo F Spencer F A model-based background adjustment for oligonucleotide expression arrays J American Stat Assoc 2004 99 909 917 10.1198/016214504000000683

[B39] Bioconductor http://www.bioconductor.org

[B40] Holm S A simple sequentially rejective multiple test procedure Scand J Statist 1979 6 65 70

[B41] Hochberg Y A sharper Bonferroni procedure for multiple tests of significance Biometrika 1988 75 800 802 10.1093/biomet/75.4.800

[B42] Sidak Z Rectangular confidence regions for the means of multivariate normal distributions J American Stat Asso 1967 62 626 633 10.2307/2283989

