# Supplementary material for: Parallel multiplicity and error discovery rate (EDR) in microarray experiments
Source: BMC Bioinformatics. 2010 Sep 16;11:465. doi: 10.1186/1471-2105-11-465 (PMC2955048; doi:10.1186/1471-2105-11-465)
Supplement: Additional file 1 — R source code for EDR method. The EDR method was implemented for two group comparison experiments either with p-values provided or without p-values. [file 1471-2105-11-465-S1.PDF]

Additional File 1

```
############
##Please cite:
##Wayne Wenzhong Xu
##Supercomputing Institute for Advanced Computational Research
##University of Minnesota

##how to Run:

##Place the data file in current directory, rename the file name as
data.txt.
##The data file is tab-delimited txt file. The first column must be
gene ids,
##second col is p-values or empty, then sequentially by groups the
expression value columns.

##function parameters: (logs,pv,g11,g22)
##if the expressions are log transformed, then logs is 1
##if p-values are provided in second column, then pv is 1 (if no pv,
need multtest library for t tests)
##group one sample number, g11
##group two sample number, g22

## example: EDR<-EDRfun(1,1,3,3) for a data file that is in log
value,
## has p-values, and two groups, each group has 3 replicates.




setwd(".")
source("EDRfun.r")
EDR<-EDRfun(1,1,3,3)


###############

EDRfun<-function(logs,pv,g11,g22){

setwd(".")


log<-logs
p<-pv
g1<-g11
g2<-g22


g12<-g1+g2
ss<-g1+1
```

```
data1<-read.table("data.txt",sep="\t", header=T)
len<-length(data1[1,])

data2<-data1[,3:len]
data2<-as.matrix(data2)
rownames(data2)<-data1[,1]

if(log == 1){
data2<-2^data2
}


if(p==1){
rawp<-data1[,2]
}else{

cl<-c(rep(0,g1),rep(1,g2))

library(multtest)
tstat<-mt.teststat(data2,cl)
rawp<-2*(1-pnorm(abs(tstat)))
}

pct0.995<-quantile(data2,0.995)
pct0.005<-quantile(data2,0.005)

data2[data2>pct0.995]<-pct0.995
data2[data2<pct0.005]<-pct0.005

med<-median(data2,na.rm=T)

rownum<-length(data2[,1])
FcFm<-matrix(0,rownum,4)
pFcFm<-cbind(rawp,FcFm)
colnames(pFcFm)<-c("rawp","Fm","Fc","c","f")
rownames(pFcFm)<- data1[,1]


for(i in 1:rownum){
maxm<-max(mean(data2[i,1:g1]),mean(data2[i,ss:g12]))
pFcFm[i,2]<-maxm/med
ratio<-mean(data2[i,1:g1]) / mean(data2[i,ss:g12])
pFcFm[i,3]<-2^abs(log(ratio,2))
x<-pFcFm[,1]
p<-1-pFcFm[i,1]
pFcFm[i,4]<-length(x[x>=p])+1
pFcFm[i,5]<- pFcFm[i,2]*(pFcFm[i,3]-1+0.000001)
}

sort_rawpM4<-pFcFm[order(pFcFm[,1]),]   ### when order, any column
include the rownames
```

```
### input: a matrix with rawp, Fold Fc, fold of max mean to median Fm
Errorr
computEDR <-function(sorted_pvs){
    data3<-sorted_pvs
    len3<-length(data3[,1])
    data3<-cbind(data3,1.0)
    colnames(data3)<-c("rawp","Fm","Fc","c","f","edr")
    x<-0
   for(i in 1:len3){
      c<-data3[i,4]
      f<- data3[i,5]
      cf<-c/f
      x<-data3[i,1]*cf
      x<-min(x,1.0)
  data3[i,6]<-x
    }
data3;
}


EDR1<-computEDR(sort_rawpM4)
EDR<-cbind(EDR1[,1],EDR1[,6])
rownames(EDR)<-rownames(EDR1)

write.table(EDR, file="EDR.txt", sep="\t")
save(EDR,file="EDR.Rdata")

return(EDR)

}
```
