# Supplementary material for: Parallel multiplicity and error discovery rate (EDR) in microarray experiments
Source: BMC Bioinformatics. 2010 Sep 16;11:465. doi: 10.1186/1471-2105-11-465 (PMC2955048; doi:10.1186/1471-2105-11-465)
Supplement: Additional file 2 — Simulation and parameters. Existing Mouse GeneChip data [23] was simulated with a different proportion (S0) of differentially expressed genes. [file 1471-2105-11-465-S2.PDF]

Simulation and parameters		
**Real data set (exprs)**		
Number of genes	m	45101
Minimum expression value	min	min(exprs)
Maximum expression value	max	max(exprs)
50 percentile	pct50	quantile(exprs,0.5)
90 percentile	pct90	quantile(exprs,0.9)
minimum SD	minsd	min(sd(t(exprs))
maximum SD	maxsd	max(sd(t(exprs))
**Settings**		
Proportionof changed genes	s0	0.001,0.003,0.005,0.01,0.02,0.05,0.1,0.2
number of DEGs	DG#	s0*m
number of non DEG	nDG#	m-DG#
sample sizes	n1	5
	n2	5
**non DEGs simulation**		
mean vector of n1	nu1	runif(nDG#,min,max)
mean vector of n2	nu2	nu2=nu1
sd vector of n1	nsd1	runif(nDG#,minsd,maxsd)
sd vector of n2	nsd2	nsd2=nsd1
gene i expression values of n1	nDn1Xi	rnorm(n1,nu1[i],nsd1[i])
gene i expression values of n2	nDn2Xi	rnorm(n2,nu2[i],nsd2[i])
**DEGs simulation**		
1.5-3 fold change generator	ff	c(runif(1000,1.5,3),runif(1000,0.333,0.6667))
fold change vector	Fc	sample(ff,DG#,replace=TRUE)
Mean vector of n1	du1	runif(DG#,pct50,pct90)
Mean vector of n2	du2	du1*Fc
sd vector of n1	dsd1	du1/5
sd vector of n2	dsd2	du2/5
gene i expression values of n1	Dn1Xi	rnorm(n1,du1[i],dsd1[i])
gene i expression values of n2	Dn2Xi	rnorm(n2,du2[i],dsd2[i])
