# Supplementary figures and images for: Parallel multiplicity and error discovery rate (EDR) in microarray experiments
Source: BMC Bioinformatics. 2010 Sep 16;11:465. doi: 10.1186/1471-2105-11-465 (PMC2955048; doi:10.1186/1471-2105-11-465)

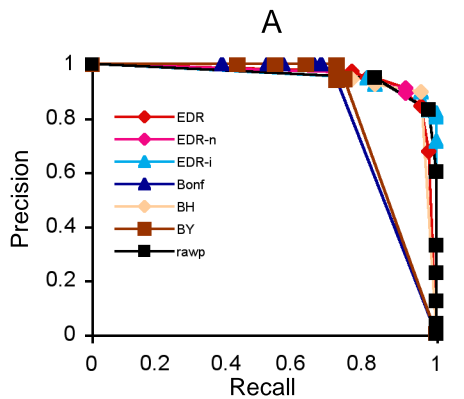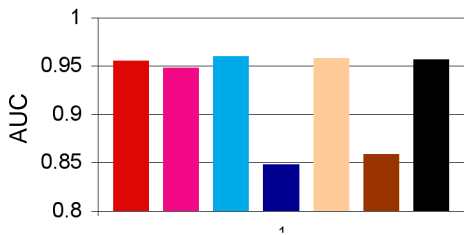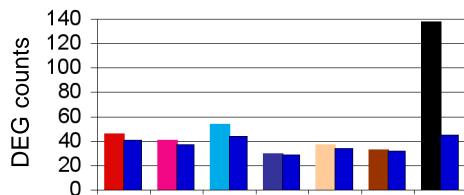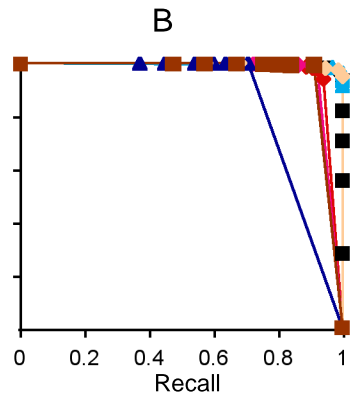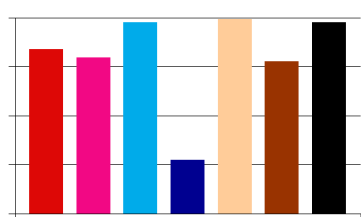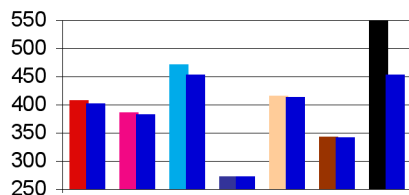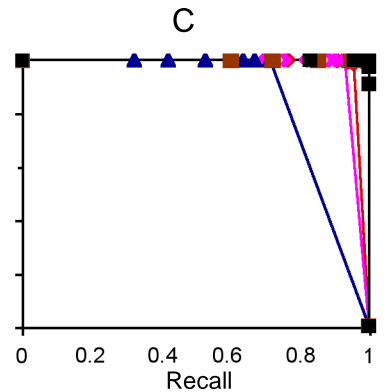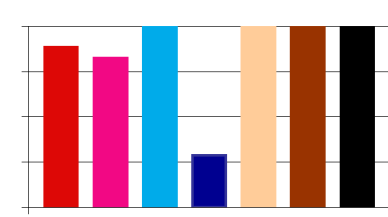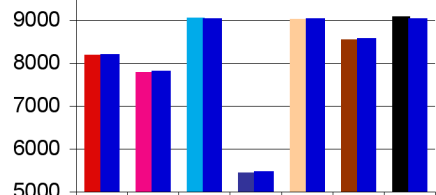

Supplement: Additional file 3 — Precision-Recall (PR) curves. Precision-Recall (PR) curves of multiple test methods on simulation data sets with different proportions of DEGs (A, S0: 0.001; B, S0: 0.02; C, S0: 0.2). Upper panel: PR curves; middle panel: the area under the curve (AUC); lower panel: simulated true DEGs (blue bars) contained within all detected DEGs of each method were at or below the significance level of 0.05. [file 1471-2105-11-465-S3.PDF]
